# Harnessing Social and Behavioural Change Strategies for the Prevention and Control of COVID-19: A Perspective from Tanzania

**DOI:** 10.21315/mjms2021.28.4.16

**Published:** 2021-08-26

**Authors:** Vivian Mushi, Lilian Mushi

**Affiliations:** 1 Department of Parasitology and Medical Entomology, School of Public Health and Social Sciences, Muhimbili University of Health and Allied Sciences, Dar es Salaam, Tanzania; 2 School of Medicine, ST. Joseph College of Health Sciences, Dar es Salaam, Tanzania

**Keywords:** social and behavioural change, COVID-19, preventive behaviour, Tanzania

## Abstract

Social and behavioural change strategies are crucial to facilitating the adoption of preventive behaviours during the COVID-19 pandemic. For the success of such social and behavioural changes, the community should be aware of the interventions and willing to adhere to health advice. This letter details the strategies employed in Tanzania to accelerate the adoption of preventive behaviour and contain the spread of COVID-19.

Dear Editor-in-Chief,

Coronavirus disease 2019 (COVID-19) is a contagious disease caused by the novel virus severe acute respiratory syndrome coronavirus 2 (SARS-CoV-2), which is transmitted from human to human via droplets, contact, airborne aerosols and fomites (1, 2). Since its outbreak in late 2019, as of 12 October 2020, it has spread to more than 235 countries or territories, with more than 119,191,045 confirmed cases and 2,643,360 confirmed deaths (3). There is currently no cure or vaccine for the disease. However, phase 2 and 3 clinical trials of potential drugs and vaccine candidates are underway (4).

To contain the spread of COVID-19, the World Health Organization (WHO) has recommended a series of preventive behavioural measures, including regular handwashing with water and soap (HWWS) or alcohol-based sanitisers, physical distancing, wearing masks, avoiding crowds and coughing into a bent elbow or tissue (5, 6). For the successful implementation of such public health measures, the WHO has stressed the need for behavioural changes at the community level. However, behavioural change is challenging because it requires individuals to abandon familiar practices and adopt potentially unfamiliar practices (7). Therefore, effective strategies are required to facilitate such changes (8).

In Tanzania, the first COVID-19 case was confirmed on 16 March 2020. Social and behavioural strategies were subsequently employed to limit the spread of the disease to other regions. Behavioural change strategies included the following:

- The government launched health promotion campaigns focusing on education and training to raise awareness of COVID-19 transmission and prevention. These included information on the benefits of preventive behaviours. The health promotion programme aimed to motivate people to adopt new behaviours and was accompanied by the installation of handwashing facilities in all public areas and the distribution of masks and hygienic kits to communities. Posters and banners emphasising the necessity and benefits of preventive measures were placed in all public areas.
- Digital communication tools were used to encourage preventive behaviours and promote new social norms. Citizens received daily reminders of the importance of HWWS, wearing masks, and physical distancing via short massage services and social media. Mass media were consistently used to provide correct information on the pandemic and to eliminate threat perceptions and panic arising from misinformation.
- A restriction strategy was implemented to influence the process of behavioural change by establishing social rules on what was and was not acceptable during the pandemic. The government set restrictions on public gatherings. Public events such as funerals were restricted to family members only, while religious activities were conducted with careful observation of preventive measures such as mask-wearing, handwashing, maintaining distances between individuals, and decontamination of surfaces, such as benches and chairs. Although it was undoubtedly difficult to adhere to such unfamiliar regulations at first, people increasingly recognised the value of the strategy when the number of new cases started to decline.
- An environmental control strategy was employed to accelerate the process of behavioural change. This strategy consisted in controlling environments that could facilitate the transmission of the disease. Events that could foster transmission, such as weddings, sports events, musical events, and political gatherings, were banned, and schools and universities were closed. This strategy helped to further control the spread of COVID-19.
- A modelling strategy was also employed to promote behavioural change. Government officials, religious leaders and celebrities served as role models of preventive behaviour in health promotion campaigns. For example, government officials were seen wearing masks, washing their hands, and bumping elbows or tapping feet instead of shaking hands. This facilitated the adoption of such behaviours by most citizens.

The implementation of behavioural change strategies has contributed to the containment of the spread of COVID-19 in Tanzania. The implemented strategies have motivated people to adopt recommended preventive behaviours, controlled environments to support behavioural change and helped change traditional social norms.

In conclusion, the social and behavioural change strategies employed in Tanzania have confirmed the importance of preventive behaviours for containing the transmission of COVID-19. The lessons learned from these strategies are that the mobilisation of financial and human resources, governmental support, community awareness and willingness to change are key to the success of behavioural change interventions.
